# Online-haemodiafiltration vs. conventional haemodialysis: a cross-over study

**DOI:** 10.1186/s12882-015-0062-0

**Published:** 2015-05-09

**Authors:** Guillaume Jean, Jean-Marc Hurot, Patrik Deleaval, Brice Mayor, Christie Lorriaux

**Affiliations:** NEPHROCARE Tassin-Charcot, 7 avenue Maréchal FOCH, 69110 Sainte Foy-les-lyon, France

**Keywords:** Online haemodiafiltration, High-flux haemodialysis, Albumin, Beta2-microglobulin, Adverse events, Phosphataemia

## Abstract

**Background:**

The main short-term advantages of haemodiafiltration (HDF) are supposedly better removal of Beta2-microglobulin (ß2-m) and phosphate, and better haemodynamic stability. The main disadvantage is higher costs. The aim of the study was to compare the clinical and biological parameters associated with HDF and high-flux haemodialysis (HD), using a cross-over design, while maintaining the same dialysis parameters.

**Methods:**

All patients on a 3 × 4 hours schedule were observed during 3 identical 6-months periods: HDF1 – HD – HDF2. The mean values for the 2 last months of each period were compared.

**Results:**

A total of 51 patients (76 % males, 45 % diabetic) with a mean age of 74 ± 15 years, and who had been on dialysis for 49 ± 60 months were included. The mean blood flow (329 ± 27 ml/min), dialysate flow (500 ml/min), and convection volumes (21.6 ± 3.2 L) were recorded. Patient medications were not changed. Predialysis blood pressure, phosphataemia, calcaemia, iPTH, Kt/V, nPNA and intradialytic events were similar throughout the 3 periods. Only serum albumin (34. 4 ± 3.6, 35.9 ± 3.4, 34.1 ± 4 g/L, p < 0. 0001) and ß2-m serum levels (26.1 ± 5.4, 28 ± 6, 26.5 ± 5 mg/L, p < 0.001, values shown for HDF1, HD, HDF2, respectively) were significantly lower during the HDF periods. Factor associated with higher delta serum albumin levels between HD and HDF periods was mainly a lower convection volume.

**Conclusion:**

Comparing HDF and HD, we did not observe any differences in haemodynamic stability or in serum phosphate levels. Only serum ß2-m (−6 % vs. HD) and albumin (−5 % vs. HD) levels changed. The long-term clinical consequences of these biochemical differences should be prospectively assessed.

## Background

In order to increase mid-to-large molecule clearance by combining diffusive and convective transport, online haemodiafiltration (HDF), using ultrapure dialysate, was introduced [1]. In the past decade, evidence has accumulated regarding the superiority of postdilution HDF over haemodialysis (HD). Specifically, HDF has been associated with higher survival rates compared with low- [2] and high-flux HD [3], when using high convection volumes as prescribed in a recent prospective study reported by Maduell et al. [4]. Additionally, HDF has been reported to provide better hemodynamic stability [4, 5], especially when using higher convective volumes [6]; a better quality of life [7]; and fewer depression symptoms [8]. HDF has also been reported to improve beta2-microglobulin (ß2-m) [2, 9], phosphate [9, 10] and urea removal [2, 9]. Some others studies have reported better anaemia correction [11] and lower inflammation [12] when using HDF. The main disadvantages of HDF are its cost [13] and the loss of albumin [14, 15].

Previously at our institution, the conventional HD protocol was 5 hours 3 times weekly using high-flux HD. In December 2009, for organizational and cost reasons, it was changed to 4 hours, and it has been hypothesized that the efficiency of postdialysis online HDF could compensate for the missed hour of therapy. The aim of the present study was to compare postdilution HDF and high-flux HD, in term of their clinical and biological parameters, using a cross-over design.

## Methods

We took the opportunity of the dialysis centre being relocated and the need for water treatment validation to interrupt HDF during 6 months allowing a cross-over follow-up.

In December 2010, all patients on a 3 × 4-hours schedule were prospectively observed during 3 × 6-months periods that included HDF1, HD and HDF2, after informed consent was obtained. The study protocol was ethical according to national standards of human experimentation and the Declaration of Helsinki. Due to lack of randomization, a local committee advice was not mandatory.

The primary objective was to compare the effect of postdilution online HDF with high-flux HD on dialysis dose, blood pressure control, intradialytic tolerance (symptomatic hypotension episodes and cramps), nutrition (dry body weight, normalized protein catabolic rate, albumin), anemia, and serum phosphate and ß2-m levels. The inclusion criteria were patients aged ≥18 years with end-stage renal disease (ESRD) receiving thrice-weekly HDF for ≥ 3 months. Exclusion criteria included active systemic diseases, liver cirrhosis, malignancies, single- needle dialysis, and use of temporary non-tunnelized catheters.

Patients were dialyzed thrice-weekly, with a 4-hour schedule, using a Fresenius 5008 console and polysulfone high-flux filter (FX80 and FX100, Fresenius S.E., Bad-Homburg, Germany). The composition of dialysate and the HDF infusate was the same throughout the 3 periods: sodium 138–140 mmol/L, potassium 2–3.0 mmol/L, calcium 1.25–1.75 mmol/L, magnesium 0.5 mmol/L, chloride 106–109 mmol/L, bicarbonate 34–37 mmol/L, acetate 3–4 mmol/L, and glucose 1.0 g/L. The dialysate calcium concentration varied from 1.25 to 1.75 mmol/l, according to the serum level of parathyroid hormone (PTH), calcium, and bone markers serum levels as reported previously [16]. Both HDF and HD were performed with ultrapure dialysis fluids. In HDF, convection volume was driven automatically using the “auto sub” system of the 5008 machine.

The blood and dialysate flow rates, and dialysate composition, were kept constant during the 3 periods. Antihypertensive medications and bone-mineral-related treatments were maintained stably.

The following parameters were recorded at baseline and at every session: dialyzer characteristics, dialysis time, blood flow rate, dialysate flow rate, vascular access, dry body weight, predialysis and postdialysis body weight, convective volume, and pre- and postdialysis systolic and diastolic blood pressures (BPs). The following laboratory data were recorded at baseline and every month: predialysis urea, creatinine, bicarbonate, sodium, brain natriuretic peptide (BNP), potassium, calcium, phosphate, intact PTH and haemoglobin. Other parameters that were recorded bimonthly included serum albumin, ß2-m and C-reactive protein levels.

Hydratation status was assessed using a body composition monitor (BCM, Fresenius Medical Care S.E., Bad-Homburg, Germany) and the postdialysis value was recorded.

Using predialysis and postdialysis urea concentrations in a mid-week dialysis session, the dialysis dose (Kt/V by Daugirdas’ second-generation single-pool, variable volume formula) and normalized protein catabolic rate (nPCR) were calculated by standard formulas. All laboratory determinations were performed locally (Grand Vallon laboratory, NOVESCIA, Lyon, France). The doses of erythropoiesis-stimulating agents, antihypertensive drugs, vitamin D, cinacalcet and phosphate binders were also recorded at baseline and every 3 months.

### Statistical analysis

Statistical analyses were carried out using MedCalc© software 11.5.1.0 (MedCalc Software, Mariakerke, Belgium). The mean biological values for the last 2 months of each period were retained for analysis. The mean values during each period, for BP, convective volume, hypotension, cramps and dry body weight, were recorded.

The differences between the 3 periods were investigated using ANOVA and a paired *t*-test. The correlations between parameters were analyzed using a Pearson’s test, when the distribution was normal, and by Spearman’s rank test if there was a non-normal distribution. Logistic regression was used when necessary. A Receiver operator analysis (ROC) was generated when necessary. Throughout the analysis, the p < 0.05 probability level was considered statistically significant. Data are presented as mean ± SD.

## Results

Among the 75 initial patients, 15 died, 6 underwent successful kidney transplantation, and 3 were lost to follow-up due to centre changes; the 51remaining patients were the subjects of the present investigation. These patients had a mean age of 74 ± 15 years, were predominantly male (76 %), included a large number of diabetics (45 %), and had been undergoing dialysis for 49 ± 60 months.

Dialysis filters were FX 80 or FX 100 polysulfone (Fresenius© Bad Homburg Germany), native AV fistulas were used in 86.5 % of patients, and the mean dialysate calcium was 1.52 mmol/L. All these parameters were stable throughout the study.

The comparison of the biological and treatment parameters, during the 3 periods, is displayed in Table 1. Throughout the study, the number of patients receiving particular types of medications remained constant throughout the 3 periods: alfacalcidol (35 %, 37 %, 37 % of patients), calcium-based phosphate binders (50 %, 48 %, 48 %), sevelamer (31 %, 31 %, 33 %), cinacalcet (5.8 %, 5.8 %, 7.8 %), antihypertensive medications (37.3 % in all 3 periods) and native vitamin D (100 % in all 3 periods). Predialysis systolic and diastolic BPs and the incidence of intradialytic symptoms were not significantly different during the 3 periods. Similarly, the dialysis dose remained stable, with mean Kt/V ranging from 1.67 ± 0.2 to 1.74 ± 0.2 Haemoglobin, transferrin saturation index, and ferritin did not differ. There were no differences in the proportion of patients treated with distinct ESA (85 %). Intravenous iron supplements (150 to 180 mg/months) and ESA doses did not differ between the periods.

**Table 1:** Comparison of biological and treatment parameters between the 3 periods

	HDF1	HD	HDF2
Albumin (g/l)	34.4 ± 3	35.9 ± 3**	34.1 ± 4
ß2-microglobumin (mg/l)	26.1 ± 5	28 ± 6*	26.5 ± 5
Calcaemia (mmol/l)	2.2 ± 0.1	2.19 ± 0.1	2.18 ± 0.1
Phosphataemia (mmol/l)	1.58 ± 0.2	1.59 ± 0.3	1.61 ± 0.2
iPTH (pg/ml)	215 ± 110	220 ± 111	245 ± 108
Kt/V	1.67 ± 0,2	1.71 ± 0.25	1.74 ± 0.25
nPCR (g/kg/d)	1 ± 0.1	1.08 ± 0,19	1.05 ± 0,19
CRP mg/l	7.5 ± 9	7 ± 8	7.7 ± 8
Hb (g/dl)	11.7 ± 1	11.8 ± 0.8	11.7 ± 0.7
BNP (pg/ml)	445 ± 478	398 ± 466	408 ± 485
sBP/dBP (mmHg)	133/61 ± 17/11	132/59 ± 18/11	134/61 ± 18/11
Dry body weight [37]	72.6 ± 11	72 ± 12	72.1 ± 12
Interdialytic weight gain [37]	1.8 ± 0.6	2 ± 0.6	2 ± 0.7
BCM OH-post (Litre)	−0.96 ± 1.4	−1.01 ± 1.5	−1.06 ± 1.5
Dialysate calcium (mmol/L)	1.51 ± 0.2	1.56 ± 0.2	1.54 ± 0.17
Blood flow rate (m/min)	330 ± 30	328 ± 27	329 ± 26
Dialysate Flow rate (mL/min)	500	500	500
Convection volume (Litre)	22.2 ± 3.3	0	21 ± 3.1
Hypotension/ cramps (% session)	15.7	12	20
Antihypertensive medications unit/day (%)	1.2 ± 0.4 (37.3)	1.2 ± 0.4 (37.3)	1.2 ± 0.4 (37.3)
ESA U/week	5000 ± 4000	4660 ± 3500	4900 ± 3500
Alfacalcidol μg/week (%)	2.2 ± 1 (37)	2.1 ± 1 (37)	2.1 ± 1 (35)
Calcium unit/d (%)	1.9 ± 2 (48)	1.88 ± 2.2 (50)	1.9 ± 2.2 (48)
Cinacalcet mg/d (%)	52 (5.8)	52 (5.8)	47 (7.8)
Sevelamer unit/d (%)	3.3 ± 4 (31)	3.4 ± 4 (33)	4 ± 4 (33)

*p < 0.001, **p < 0.0001 vs. other periods (ANOVA)

**Fig. 1:**
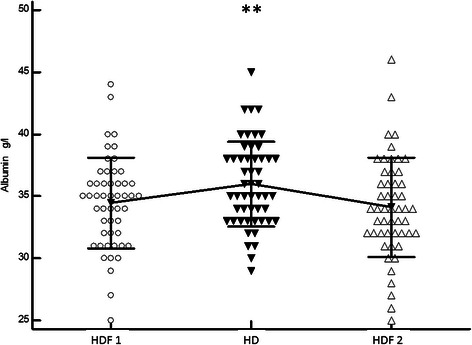
Comparison of serum albumin value between the 3 periods (Paired *t*-test): Alb HDF1 (34.4 ± 3 g/L) vs. Alb HD (35.9 ± 3 g/L), mean difference 1.5 ± 2.5 g/L, p = 0.0001; Alb HD vs. Alb HDF2 (34.1 ± 3.9 g/L) mean difference 1.8 ± 2.6 g/L, p < 0.0001; Alb HDF1 vs. HDF2, mean difference 0.3 ± 3 g/L, p = 0.43

**Fig. 2:**
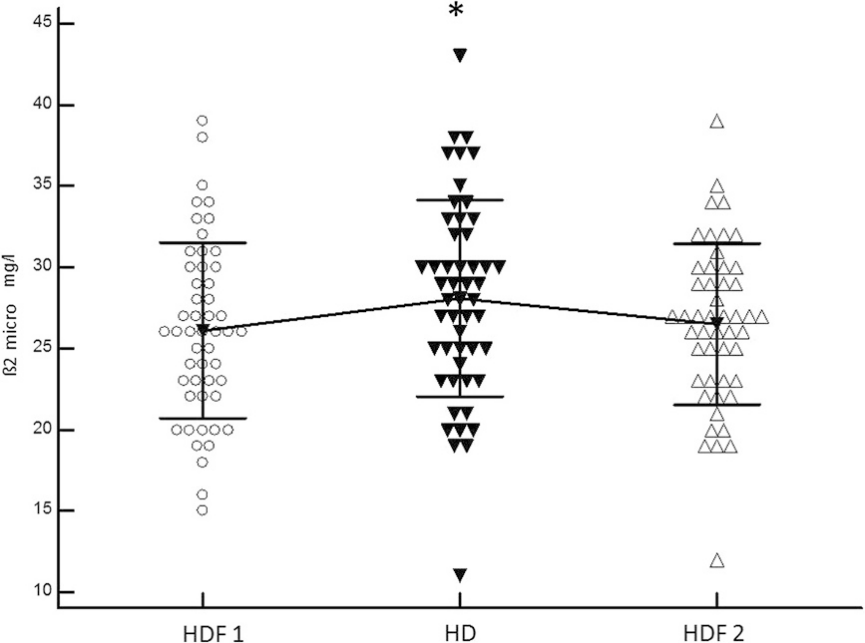
Comparison of serum ß2-m values between the 3 periods (Paired *t*-test): ß2-m HDF1 (26.1 ± 5 mg/L) vs. ß2-m HD (28 ± 6 mg/L), mean difference 1.9 ± 3.6, p = 0.0003; ß2-m HD vs. ß2-m HDF2 (26.5 ± 4.9 mg/L) mean difference 1.5 ± 3, p = 0.0009; ß2-m HDF1 vs. HDF2, mean difference 0.3 ± 2.7 mg/L, p = 0.37

Only serum albumin concentrations (34. 4 ± 3.6, 36 ± 3.4, 34.1 ± 4 g/L, p < 0. 0001, Fig. 1) and ß2-m serum levels (26.1 ± 5.4, 28 ± 6, 26.5 ± 5 mg/L, p < 0.001, Fig. 2) were significantly different during the 3 periods (HDF1, HD, HDF2). Compared with HD, 31/51 patients (60 %) had lower serum albumin levels during HDF1 (Δ -1 to −10 g/L) and 26/51 patients (50.9 %) displayed lower albumin levels during both HDF periods. Factors associated with higher Δ serum albumin levels during the HD and HDF periods included diabetes (64 % vs. 23 %, p = 0.04), lower serum albumin in HDF1 (33.2 ± 3.9 vs. 35.7 ± 3 g/L, p = 0.01) and lower convection volumes (20.8 ± 2.5 vs. 22.9 ± 2.4 L, p = 0.04). Convection volume was similar in diabetic and non-diabetic patients (21.3 ± 3.8 vs. 21.9 ± 2.8 L). The serum albumin level in HDF1 was similar in diabetic and non-diabetic patients (33 ± 2.8 vs. 34.4 ± 3.7 g/L).

**Fig. 3:**
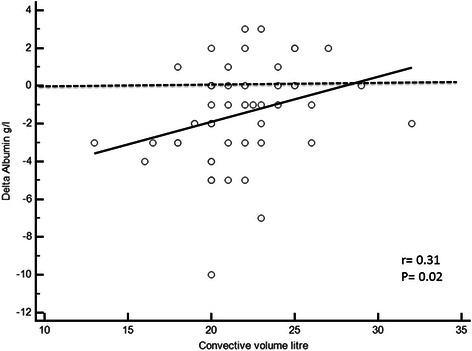
Linear regression between convective volume in HDF1 and delta albumin between HD and HDF1 periods

**Fig. 4:**
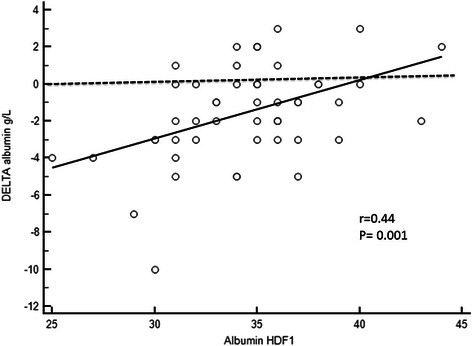
Linear regression between serum albumin in HDF1 and delta albumin between HD and HDF1 periods

Delta albumin demonstrated inverse relationship with convective volume (Fig. 3) and albumin in HDF1 period (Fig. 4). However, using logistic regression, only low convective volume remained associated with higher delta albumin (Table 2). The best cut-off value was 21 L, with a specificity of 76.9 % and a sensitivity of 64 % (Area under the curve = 0.75 [0.607 to 0.859], p = 0.004).

**Table 2:** Logistic regression of factors associated with positive delta serum albumin between HDF1 and HD period

Variable	Coefficient	Std. error	P	Odds ratio	95 % CI
Convective volume L	−0.25135	0.11949	0.0354	0.7777	0.6154 to 0.9830
Albumin g/L (HDF1)	−0.20410	0.10276	0.0470	0.8154	0.6666 to 0.9973
Diabetes	0.16828	0.58480	0.7735	1.1833	0.3761 to 3.7229

Also during the study, 31/51 (60.7 %) patients had lower serum ß2-m values during HDF1 than during HD, and 32/51 (62.7 %) had lower ß2-m values in HDF2 than during the HD period (Fig. 2). The only factor associated with the absence of ß2-m decline during the HDF periods was diabetes (72 % vs. 28 %, p = 0.01).

## Discussion

In this cross-over study comparing 3 consecutive of 6-month periods (HDF1 − HD − HDF2), we found that HDF protocols were associated with lower albumin and ß2-m serum levels. No others clinical or biological parameters were found to differ during the 3 periods. A convective volume < 21 L was associated with the lower albumin levels during HDF.

In a 4-years observational study in 2006, slight decreases in serum albumin and prealbumin levels were reported during the first 6 months following a switch from HD to HDF with increased levels observed thereafter [17]. Ok et al. also reported lower albumin levels in the low-efficiency HDF arm of their prospective Turkish study [3]. Decreased serum albumin levels during the first 6 months have also been reported after switching from high-flux HD to HDF [18]. However, these observations were not confirmed in a study in Balkan countries [19], and Movilli et al. reported the absence of an albumin level decrease during an HDF protocol [9].

The lower serum albumin level observed during the HDF periods in the present study could be due to a dialysate albumin loss, as reported previously by Combarnous et al., who observed an albumin loss of 1000–6800 mg/session [15]. In 2004, Ahrenholz et al. reported a total albumin loss of 300–7000 mg/session, depending on the type of dialyzer used [14]. Unfortunately, we did not measure the albumin loss in the dialysate. In the CONTRAST study, de Hoedt et al. reported no difference in the rate of change in albumin between the HDF and low-flux HD arms [20]. However, only annual data was reported, and short-term evolution could have been missed. As serum albumin decreased mainly in patients with lower convection volume, the relationship between the change in albumin levels and the HDF technique itself remains unclear.

A decrease in ß2-m serum values during HDF was reported by Zehnder et al., who demonstrated its absorption to the polysulfone membrane [10]. Others studies have also reported a significant decrease in ß2-m levels after switching patients from HD to HDF [9, 21, 22]. However, in the Turkish study, there were not differences between the 3 arms involving HDF and low- and high-flux HD [3]. In the CONTRAST study, ß2-m decreased significantly in the HDF arm, especially in cases when residual kidney function was low [2]. Lastly, in the ESHOL study, ß2-m levels were similar between the HD and HDF arm [4]. A role for residual renal function has been hypothesized to account for this non-expected evolution. However, the factors associated with ß2-m decline during HDF have not been clearly reported. Our observation that diabetes was associated with smaller serum ß2-m decline requires further confirmation and explanation.

The impact of serum ß2-m on the survival in haemodialysis patients is controversial [23, 24] and depends on the patient’s nutritional factors and diabetic status; however, the negative association of low albumin levels with poor outcomes is clear [25]. Thus, a negative impact of HDF on serum albumin would be expected to associate with poor outcomes, but this effect has not been reported in the literature. Even if the question of a survival advantage for HDF is still under debate, the data do not confirm any disadvantage for HDF compared with HD.

HDF was not observed to have any impact on mean phosphate level or on phosphate binder requirements in our study. The literature on this subject remains controversial. In 1999, Zenhder et al. reported an increase in phosphate clearance during HDF vs. high-flux HD [10]. In 2010, another group reported lower serum phosphate levels in HDF-treated patients, but data on protein intake or phosphate binders were not provided [26]. Lornoy et al. reported higher phosphate removal during HDF when compared with HD, especially in the low-normal phosphate range, but not in cases where phosphate levels were high [27]. Others studies have also reported decreased phosphate levels during HDF vs. HD [9, 28–30], mostly using higher blood flow rates. However, in 1991, Man et al. reported no advantage of HDF for phosphate mass transfer [23], and Ok et al. reported no differences in phosphate levels between HD and HDF groups [3]. Our observation confirms that the impact of HDF on phosphate levels is not significant. Moreover, we did not observed any changes in serum levels of PTH or bone markers, unlike a previous study that demonstrated, reductions in both PTH and bone alkaline phosphatase after switching from HD to HDF [31]. However, calcium mass transfer, a potentially key point, was not evaluated as in our study. Regarding small molecule clearance, we did not find differences between periods similar to Zehnder et al. who reported no advantage of HDF for urea and creatinine clearance [10]. By contrast, Movilli et al. [9] and the CONTRAST study [2] reported an increase in dialysis dose when using HDF vs. low-flux HD, but higher blood flow rates in the HDF arm may have biased the results.

We could not confirm any hemodynamic advantage of HDF, as we did not observe any difference in the number of hypotensive episodes or changes in blood pressure values during the 3 phases of the study. These finding are not consistent with results from postdilution HDF [5] and predilution HDF [32] studies. Additionally, the ESHOL study reported fewer hypotensive episodes in the HDF arm [4], especially when higher convective volumes were achieved, as reported by Mora-Bravo et al. [6]. However, the absence of a haemodynamic advantage for HDF has also been reported under strictly controlled conditions in 12 stable patients [33]. Similarly, no difference in blood pressure was found for HDF in association with intracellular or extracellular volume changes during sessions [34]. The favourable impact of HDF on haemodynamic stability is hypothesized to be due to higher sodium mass transfer in some cases, even if this phenomenon has been poorly documented.

We could not confirm better control of anaemia during HDF, as reported by Vilar et al. in a large observational study [35], or the correction of anaemia with reduced dose of ASE observed 9 months after switching 32 patients to HDF [11]. We also did not find any significant changes in CRP levels during the 3 periods in contrast to the results from a large multicenter crossover study [36] and 2 observational studies [5, 12].

Our study has numerous limitations including that the initial dialysis technique was not randomized, the size of the studied cohort was small, and we did not measure albumin loss in the dialysate. However, the cross-over design of the study could give a more sound comparison of the 2 modalities. Nevertheless, there was also some drop out and the patients’ state may have changed after 6 or 12 months.

## Conclusion

Comparing online postdilution HDF and high-flux HD in a cross-over study, we observed no differences in patient haemodynamic stability, anaemia, inflammation or serum phosphate levels. Only serum ß2-m (−6 % vs. HD) and albumin (−5 % vs. HD) levels were significantly different. The long-term clinical impact of these biochemical observations, and their association with the convective volume should be prospectively assessed.

## Abbreviation

ß2-m: Beta2-microglobulin
BCM: Body composition monitor
BNP: Brain natriuretic peptide
BP: Blood pressure
CRP: C-reactive protein
ESRD: End stage renal disease
ESA: Erythropoiesis stimulating agent
HD: Haemodialysis
HDF: Haemodiafiltration
nPCR: Normalized protein catabolic rate
PTH: Parathyroid hormone
